# Generation of stable PDX derived cell lines using conditional reprogramming

**DOI:** 10.1186/s12943-017-0745-1

**Published:** 2017-12-06

**Authors:** Alexandra Borodovsky, Travis J. McQuiston, Daniel Stetson, Ambar Ahmed, David Whitston, Jingwen Zhang, Michael Grondine, Deborah Lawson, Sharon S. Challberg, Michael Zinda, Brian A. Pollok, Brian A. Dougherty, Celina M. D’Cruz

**Affiliations:** 1 Bioscience, Oncology, IMED Biotech Unit, AstraZeneca, Boston, USA; 2 Propagenix Inc, 9605 Medical Center Drive #325, Rockville, MD 20850 USA

**Keywords:** Patient derived xenograft, Conditional reprogramming, Cell line models, Drug discovery, Oncology

## Abstract

Efforts to develop effective cancer therapeutics have been hindered by a lack of clinically predictive preclinical models which recapitulate this complex disease. Patient derived xenograft (PDX) models have emerged as valuable tools for translational research but have several practical limitations including lack of sustained growth in vitro. In this study, we utilized Conditional Reprogramming (CR) cell technology- a novel cell culture system facilitating the generation of stable cultures from patient biopsies- to establish PDX-derived cell lines which maintain the characteristics of the parental PDX tumor. Human lung and ovarian PDX tumors were successfully propagated using CR technology to create stable explant cell lines (CR-PDX). These CR-PDX cell lines maintained parental driver mutations and allele frequency without clonal drift. Purified CR-PDX cell lines were amenable to high throughput chemosensitivity screening and in vitro genetic knockdown studies. Additionally, re-implanted CR-PDX cells proliferated to form tumors that retained the growth kinetics, histology, and drug responses of the parental PDX tumor. CR technology can be used to generate and expand stable cell lines from PDX tumors without compromising fundamental biological properties of the model. It offers the ability to expand PDX cells in vitro for subsequent 2D screening assays as well as for use in vivo to reduce variability, animal usage and study costs. The methods and data detailed here provide a platform to generate physiologically relevant and predictive preclinical models to enhance drug discovery efforts.

**Electronic supplementary material:**

The online version of this article (10.1186/s12943-017-0745-1) contains supplementary material, which is available to authorized users.

## Main text

Despite advances in our understanding of the etiology and pathogenesis of cancer, the failure rate of novel therapies remains high. Current drug discovery efforts have predominantly utilized standard cancer cell lines due to their ease of proliferation and amenability to numerous preclinical applications. While these models have yielded key insights into mechanisms of malignant transformation and drug response, recent data suggests that the selective pressure of cell culture leads to a divergence from the tumors from which they were derived [1]. Therefore, there is a need for more physiologically relevant and clinically predictive models of human cancer to advance drug discovery and development.

Patient-derived xenografts (PDX) - in which patient tumor and stromal tissue is implanted directly into immunocompromised animals and maintained in vivo *-* have emerged as important tools for preclinical and translational research. PDX models have several advantages over standard cell line xenografts including preservation of gene expression patterns, continuance of tissue histology and maintenance of chromosomal architecture. Despite their benefits, PDX models are limited by several factors including slow growth, variable engraftment rate and lack of sustained growth in vitro. While PDX cells may be grown in 2D and 3D cultures for short periods of time, most PDX cell lines cease proliferating and undergo senescence within several passages [1].

**Fig. 1:**
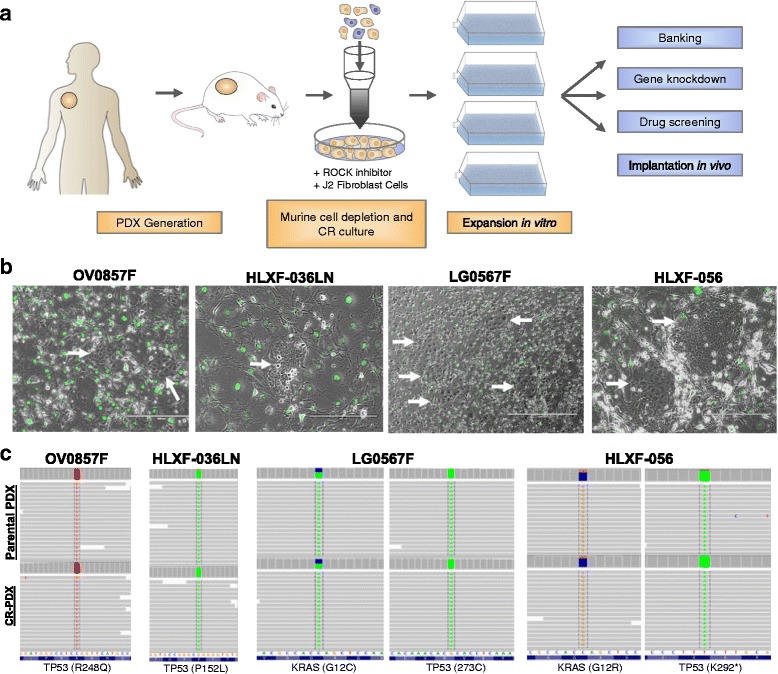
CR technology expands PDX derived cell cultures and maintains genetic profile of the parental PDX tumor. **a** Experimental design for the establishment of PDX cell lines **b** Representative images of PDX cells growing in co-culture with GFP-expressing 3 T3-J2 cells (arrows). OV0857F (10X), HLXF-036LN (10X), LG0567F (4X), HLXF-056 (10X). **c** Targeted genetic sequencing revealed that the CR-PDX cell lines maintain key mutations and allele frequency of the parental PDX

Recently, Conditional Reprogramming (CR) technology has been successfully used to achieve sustained expansion of human normal and tumor epithelial cells. This method is based on co-culture of epithelial cells with growth-arrested mouse 3 T3-J2 fibroblast feeders in the presence of a Rho kinase inhibitor [2]. Epithelial cells grown under CR conditions maintain genomic stability and exhibit many properties of adult basal stem cells, but do not express markers of pluripotency such as Oct4 or Sox2 [3]. Recently, these methods have also been used to expand and characterize cancer stem cells [4]. Importantly, cell proliferation by the CR method is reversible, with removal from the growth-stimulating conditions generally leading to differentiation of these stem-like cells to their committed fate [3]. Primary tumor cells expanded in CR cultures have been shown to maintain the characteristics of the parental tumor sample, thereby enabling in vitro chemosensitivity screening assays for personalized drug profiling [5]. Here we describe a method for using CR technology to generate and expand stable PDX cell lines for in vitro and in vivo applications. The PDX tumors were enzymatically disassociated, depleted of murine cells, and CR cultures were established and expanded into stable CR-PDX lines for use in preclinical applications (Fig. 1a).

## CR technology establishes stable PDX cell lines and maintains genetics of parental tumors

The PDX tumors used in this study were originally established through subcutaneous implantation of patient tumors into immune-deficient NSG mice and consecutively passaged in vivo. Cryopreserved PDX tumors were enzymatically disassociated to form a single cell suspension and co-cultured with irradiated 3 T3-J2 feeder cells in Conditional Reprogramming Media (CRM). The number of viable cells recovered following dissociation varied greatly, owing to variations in total starting biomass and viability of the sample prior to processing (Additional file 1: Table S1). Despite relatively small starting cell numbers, the PDX models formed discrete colonies in the CR conditions (Fig. 1b). Early low-pass whole genome sequencing analysis of the CR-PDX cell lines revealed that the cultures contained a substantial percentage of murine cells, likely from the murine stroma which naturally replaces human stromal tissue within several passages. To reduce murine cell populations, CR-PDX cell cultures were subjected to magnetic mouse cell depletion. Following cell depletion, all established CR-PDX cell lines maintained steady rates of proliferation in CR conditions with varying doubling times and cellular morphologies (Fig. 1b, Additional file 1: Table S1).

A major criticism of standard cancer cell lines is that they do not accurately represent the genetics and heterogeneity of primary malignancy and are therefore limited in their usefulness in translational medicine [3]. To determine if CR-PDX cells maintain the parental mutations driving tumorigenesis, targeted sequence analyses were performed using a panel of 300 genes on late passage CR-PDX cell lines. CR-PDX lines faithfully maintained allele frequency of the parental driver mutations and no clonal drift was detected, demonstrating the genetic stability of CR-PDX cultures (Fig. 1c, Additional file 2: Figure S1).

## CR-PDX cell lines can be used for in vitro drug screening and genetic knockdown studies

**Fig. 2:**
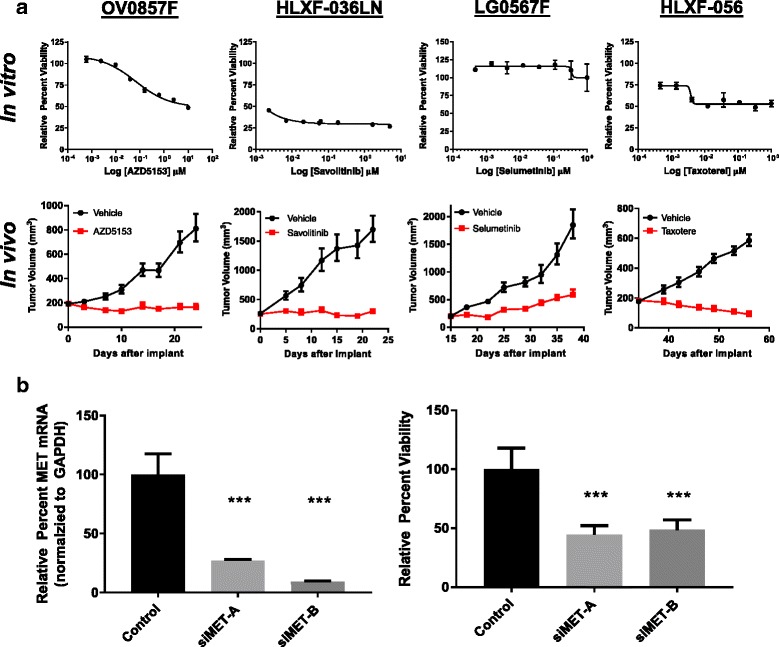
CR-PDX cell lines are amenable to in vitro preclinical applications. **a** CR-PDX cells were removed from CR conditions and treated with the indicated inhibitors (*n* = 3, mean ± SD shown). In vitro drug sensitivity of CR-PDX cells was compared to in vivo response of the parental tumor. Means ± error are shown for each model. *N* = 8 (OV0857F), *n* = 8 (HLXF-036LN), *n* = 9 (LG0567F), and *n* = 10 (HLXF-056) (**b**) CR-PDX cells are amenable to gene knockdown studies using siRNA. CR-HLXF-036LN cells were reverse transfected with indicated siRNAs for 72 h. Knockdown efficiency was confirmed by qPCR and the effect of knockdown on viability was assessed at the end of study, (*N* = 3, mean ± SD shown, ***p < 0.05, Student’s t-test, two-tailed)

We next sought to determine if the CR-PDX lines could be used for in vitro drug sensitivity screens and if so, whether the cells maintained sensitivity to targeted inhibitors in vitro as observed by the parental PDX tumor in vivo. CR-PDX cells were removed from CR conditions and treated with targeted agents with known in vivo response at concentrations ranging from 0.01-10 μM. Staurosporine was used as a positive control and viability was assessed after 3 to 5 days (Fig. 2a, Additional file 2: Figure S2). All evaluated CR-PDX cell lines were amenable to in vitro drug sensitivity screens (Fig. 2a). In in vivo efficacy studies, the BRD4 amplified OV0857F model exhibits sensitivity to BRD4 inhibitor AZD5153 with an overall tumor growth inhibition (TGI) greater than 100% [6]. Sensitivity to BRD4 inhibition is maintained in the CR-OV0857F cell line to AZD5153 (IC_50_ = 66.3 nM) as well as to JQ1 (IC_50_ = 57.1 nM) (Additional file 2: Figure S2). Similarly, the MET-dependent non-small cell lung cancer (NSCLC) HLXF-036LN PDX model is sensitive to targeted MET inhibition by savolitinib (volitinib, AZD6094, HMPL504) in vivo (TGI > 100%) as previously described [7] and the CR-HLXF-036LN cell line was sensitive to savolitinib in vitro at all doses tested (IC_50_ < 2 nM). Conversely, the KRAS (G12C) NSCLC LG0567F model is moderately sensitive to MEK inhibition by selumetinib (AZD6244, ARRY-142886) [8] in vivo (TGI = 75%) but this sensitivity is lost in vitro (IC_50_ > 1 μM). Similarly, the KRAS (G12R) NSCLC HLXF-056 model is exquisitely sensitive to taxotere in vivo (TGI > 100%) but sensitivity is lost in vitro*.*

Differences in drug sensitivity between in vitro and in vivo conditions have been documented in conventional cell lines and may stem from the loss of neighboring stromal cells in purified cultures. Recent studies have demonstrated the contribution of stromal cells within the tumor microenvironment in the control of drug sensitivity [9]. Paracrine signaling by stromal derived proteins such as WNT16B and HGF influence sensitivity to chemotherapy and targeted BRAF inhibitors in numerous cell types. While this study was not powered to fully interrogate the prevalence of in vivo*/*in vitro disconnect among PDX cell lines, additional efforts to generate and characterize CR-PDX cell lines will inform the frequency of this effect. For models dependent on stromal paracrine signaling, it may be possible to restore essential pathways using 3D scaffold cultures. Collectively, these studies show that CR-PDX cell lines are amenable to high throughput drug sensitivity screening but that an in vivo*/*in vitro disconnect may exist in some cases, though this is likely dependent on both cell line and drug mechanism of action.

One advantage of standard cell lines is the ability to investigate genotype-phenotype relationships by modification of gene expression via targeted deletion or gene knockdown. To examine if CR-PDX cell lines could be utilized for genetic knockdown studies, CR-PDX cells were transfected with targeted or scrambled control siRNAs and knockdown was confirmed by qPCR. Effect of knockdown on viability was assessed at the end of study. Targeted knockdown of MET in CR-HLXF-036LN significantly decreased cell viability, consistent with in vitro and in vivo findings (Fig. 2b). Similarly, siRNA knockdown of BRD4 decreased viability in CR-OV0857F similar to small molecule inhibition of BRD4 with AZD5153 (Additional file 2: Figure S3). These data demonstrate that CR-PDX cells can be utilized for gene manipulation studies to interrogate cell physiology and enable proof of mechanism studies in clinically relevant cell populations.

## CR-PDX cell lines can be utilized for in vivo efficacy studies

**Fig. 3:**
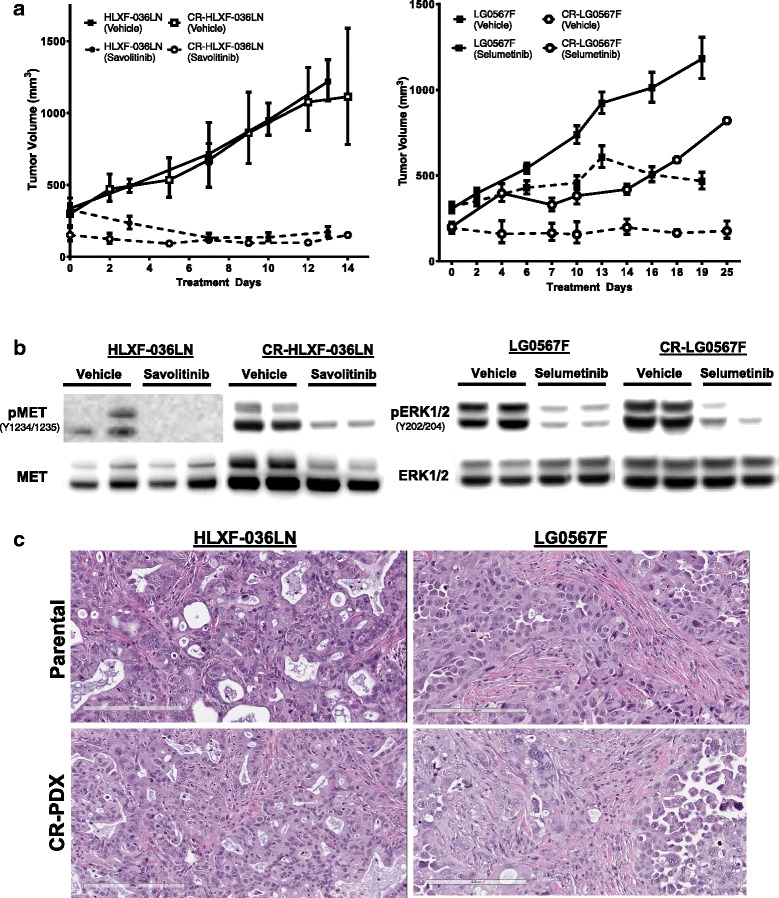
CR-PDX cell lines are amenable to in vivo preclinical applications. **a**. Re-implanted CR-HLXF-036LN and CR-LG0567F cells formed tumors in NSG mice (open symbols) and maintained growth kinetics of parental tumor (closed symbols). CR-HLXF-036LN maintained sensitivity to Savolitinib (25 mg/kg, QD, *n* = 2) and CR-LG0567F maintained sensitivity to Selumetinib (25 mg/kg, BID, n = 3) similar to parental tumors (dotted lines, n = 10 for HLXF-036LN and n = 9 for LG0567F). Mean ± error shown for each model. **b** Implanted CR-PDX tumors maintain pharmacodynamic response to targeted agents, similar to parental tumors. **c** Subcutaneously implanted CR-PDX cells produce tumors with histology similar to parental PDX tumor. Representative images shown (n = 2)

An inherent limitation of PDX models is that tumor grafts are maintained exclusively in mice requiring large animal numbers and high associated costs. Additionally, PDX propagation is conventionally performed by implantation of tumor fragments with unknown cell number and viability which leads to high inter- and intra-study variability. To determine if CR technology could address these limitations, CR-HLXF-036LN and CR-LG0567F cells were implanted subcutaneously into immunocompromised animals. Rate of tumor establishment and growth kinetics were compared to parental PDX. Both CR-PDX models formed tumors in all animals within 4 weeks of implantation, similarly to parental PDX models (Fig. 3a). Additionally, we sought to determine whether re-implanted CR-PDX tumors maintained similar drug sensitivity as parental tumor upon treatment with targeted inhibitors*.* Following establishment, mice bearing CR-HLXF-036LN tumors were treated with savolitinib (25 mg/kg, QD) and mice bearing CR-LG0567F tumors were treated with selumetinib (25 mg/kg, BID). CR-HLXF-036LN maintained identical growth kinetics and drug sensitivity to parental tumors. Following treatment with savolitinib, both parental and CR-HLXF-036LN exhibited complete tumor growth inhibition and intratumoral inhibition of MET phosphorylation (Fig. 3a left panel and Fig. 3b). Interestingly, although CR-LG0567F cells did not exhibit sensitivity to selumetinib in in vitro drug screens (Fig. 2a), drug sensitivity was restored in vivo*,* demonstrating tumor growth inhibition and pharmacodynamic modulation of pERK1/2 (Fig. 3a right panel and Fig. 3b) similar to parental LG0567F tumors. This underscores the contribution of stromal cells to signaling pathways in KRAS mutant malignancies [10]. Of note, CR-LG0567F had slightly protracted initial growth kinetics compared to parental. We hypothesize that this may be due to differences in implanted cell number and viability given that the parental PDX tumors are implanted by fragment passage so starting cell numbers and viability were unknown. Optimization of CR-LG0567F implantation number may therefore improve growth kinetics. We also observed that CR-PDX tumors maintained striking histological similarity to parental tumors including the formation of mucinous acini in parental and CR-HLXF-036LN tumors and poorly differentiated cells in the parental and CR-LG0567F tumors (Fig. 3c).

## Conclusions

Conditional Reprogramming (CR) technology can be used to generate and expand stable cell lines from PDX tumors without compromising fundamental biological properties of the model. This method offers the ability to expand PDX cells in vitro for subsequent 2D screening assays as well as for use in vivo to reduce variability, animal usage and study costs. The methods provided here provide a platform to generate physiologically relevant and predictive preclinical models to enhance drug discovery efforts.

## Additional files

Additional file 1: Table S1. PDX Model Characteristics. (DOCX 12 kb)

Additional file 2: Figure S1. SNP Fingerprinting of parental and conditionally reprogrammed samples reveals homology across all sample pairs. Sample phylogeny shows clear concordance with key SNPs, confirming sample identities. Phylogenic clustering was used to identify matched parental and conditionally reprogrammed (CR) samples. **Figure S2.** CR-PDX cells are amenable to in vitro chemosensitivity screening. CR-PDX cells were removed from CR conditions and treated with targeted agents at concentrations ranging from 0.01-10 μM. Staurosporine was used as a positive control and viability was assessed after 3 to 5 days (a) CR-OV0857F, (b) CR-LG0567F (c) CR-HLXF-036LN, (d) CR-HLXF-056. **Figure S3.** CR-PDX cells are amenable to gene knockdown studies using siRNA. CR-OV0857F cells were reverse transfected with indicated siRNAs for 120 h. Knockdown efficiency was confirmed by qPCR and the effect of knockdown on viability was assessed at the end of study. (PPTX 3628 kb)

## Abbreviations

BID: “bis in die” (twice a day)
CR: Conditional reprogramming
NSCLC: Non-small cell lung cancer
NSG: NOD.Cg-Prkdc^scid^Il2rg^tm1Wjl^/SzJ
PDX: Patient derived xenograft
QD: “quaque die” (once a day)
qPCR: Quantitative polymerase chain reaction
siRNA: Small interfering RNA
TGI: Tumor growth inhibition
