# Supplementary material for: Generation of stable PDX derived cell lines using conditional reprogramming
Source: Mol Cancer. 2017 Dec 6;16:177. doi: 10.1186/s12943-017-0745-1 (PMC5719579; doi:10.1186/s12943-017-0745-1)
Supplement: Supplementary file 1 — PDX Model Characteristics. (DOCX 12 kb) [file 12943_2017_745_MOESM1_ESM.docx]

| **PDX Model Name** | **Indication** | **WHO Grade** | **Parental Mutations** | **Starting cell number /viability (%)** | **CR-PDX doubling Time/Day** |
| --- | --- | --- | --- | --- | --- |
| HLXF-036LN | Metastatic lung Adenocarcinoma | Grade III | TP53 (P152L) | 5.9x10^4^ (5%) | 0.54 |
| HLXF-056 | Lung Adenocarcinoma | Grade II | KRAS (G12R)  TP53 (K292*) | 7.9x10^5^ (29%) | 0.16 |
| LG0567F | Lung Adenocarcinoma | Grade III | KRAS (G12C)  TP53 (R273C) | 6.27x10^5^ (32%) | 0.37 |
| OV0857F | Metastatic ovarian serous adenocarcinoma | Grade III | P53 (R248Q) | 2.52x10^5^ (32%) | 0.27 |
