# Supplementary material for: Generation of stable PDX derived cell lines using conditional reprogramming
Source: Mol Cancer. 2017 Dec 6;16:177. doi: 10.1186/s12943-017-0745-1 (PMC5719579; doi:10.1186/s12943-017-0745-1)
Supplement: Supplementary file 2 — SNP Fingerprinting of parental and conditionally reprogrammed samples reveals homology across all sample pairs. Sample phylogeny shows clear concordance with key SNPs, confirming sample identities. Phylogenic clustering was used to identify matched parental and conditionally reprogrammed (CR) samples. Figure S2. CR-PDX cells are amenable to in vitro chemosensitivity screening. CR-PDX cells were removed from CR conditions and treated with targeted agents at concentrations ranging from 0.01-10 μM. Staurosporine was used as a positive control and viability was assessed after 3 to 5 days (a) CR-OV0857F, (b) CR-LG0567F (c) CR-HLXF-036LN, (d) CR-HLXF-056. Figure S3. CR-PDX cells are amenable to gene knockdown studies using siRNA. CR-OV0857F cells were reverse transfected with indicated siRNAs for 120 h. Knockdown efficiency was confirmed by qPCR and the effect of knockdown on viability was assessed at the end of study. (PPTX 3628 kb) [file 12943_2017_745_MOESM2_ESM.pptx]

## Slide 1
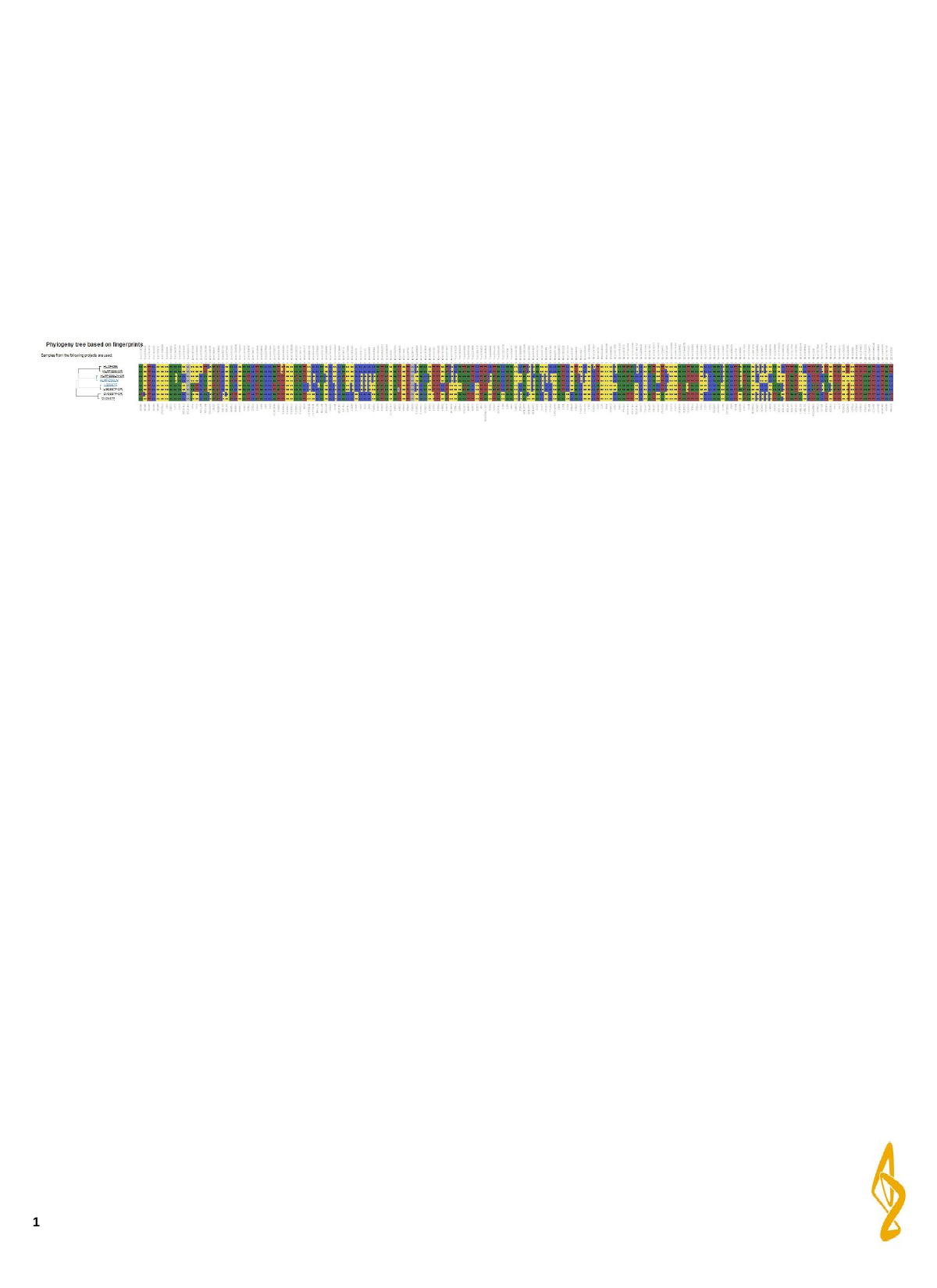

1

## Slide 2
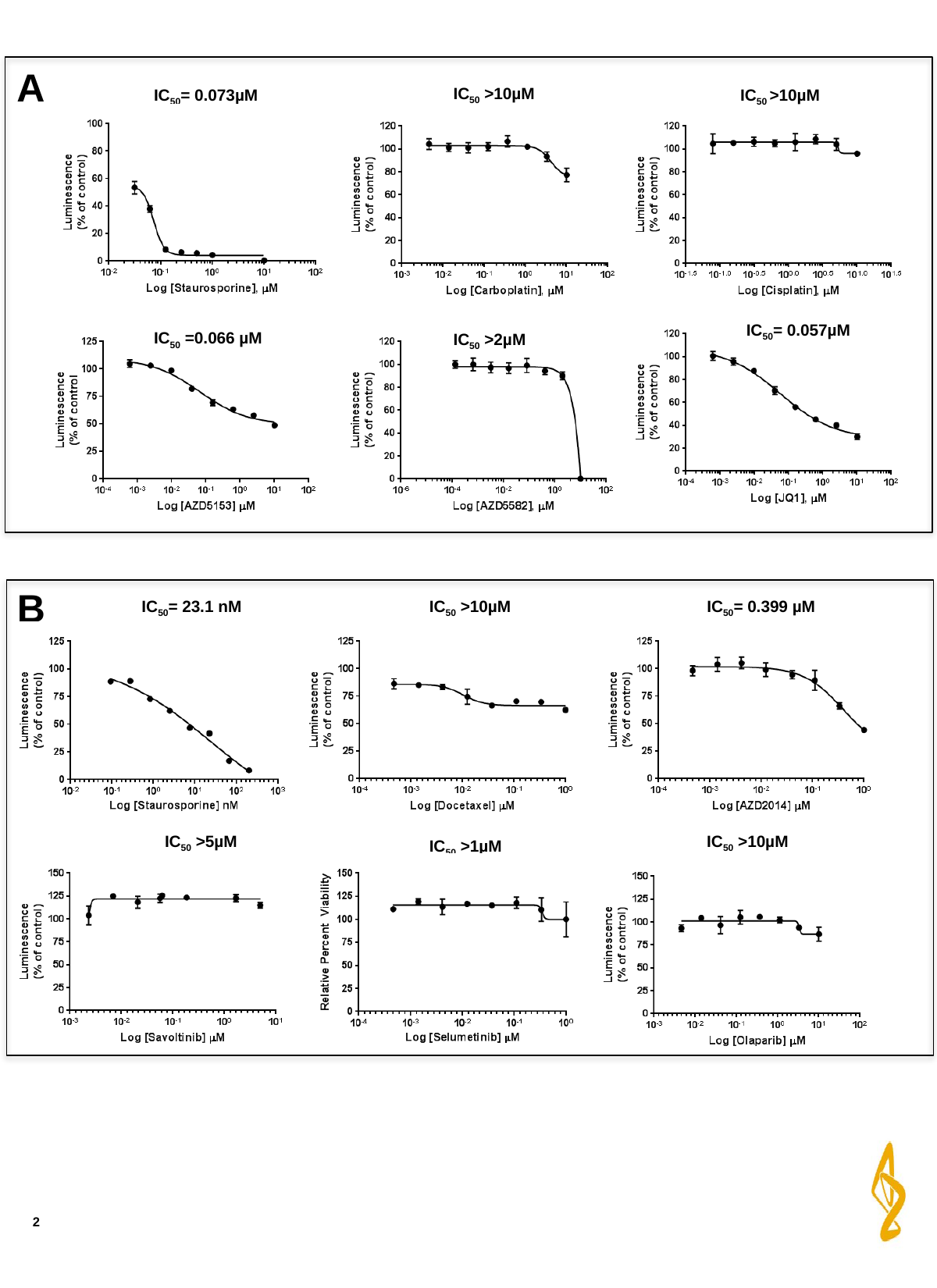

A
IC50 >10µM
IC50= 0.073µM
IC50 >10µM
IC50= 0.057µM
IC50 =0.066 µM
IC50 >2µM
B
IC50= 23.1 nM
IC50 >10µM
IC50= 0.399 µM
IC50 >5µM
IC50 >10µM
IC50 >1µM
2

## Slide 3
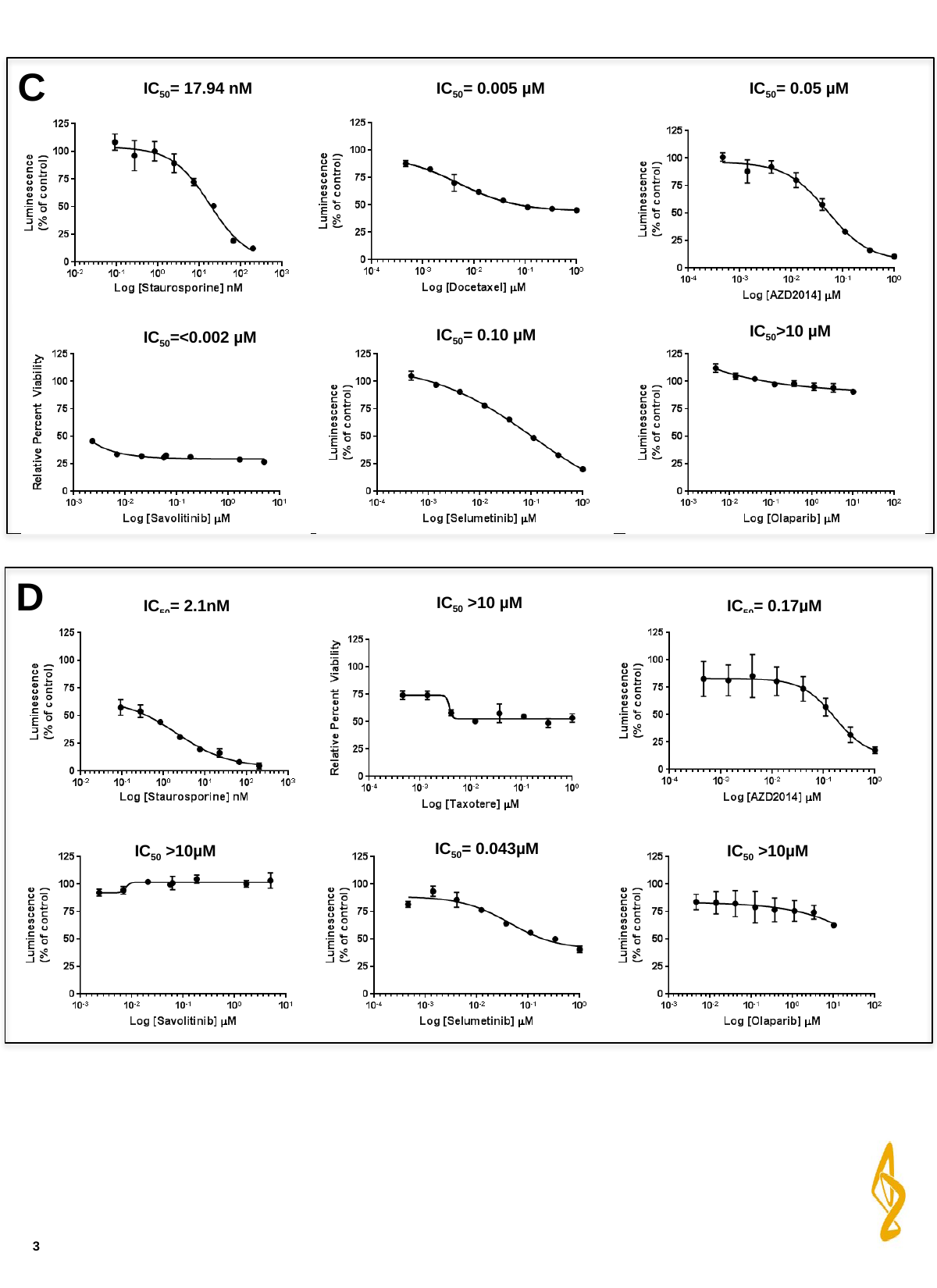

C
IC50= 17.94 nM
IC50= 0.005 µM
IC50= 0.05 µM
IC50>10 µM
IC50= 0.10 µM
IC50=<0.002 µM
D
IC50 >10 µM
IC50= 2.1nM
IC50= 0.17µM
IC50= 0.043µM
IC50 >10µM
IC50 >10µM
3

## Slide 4
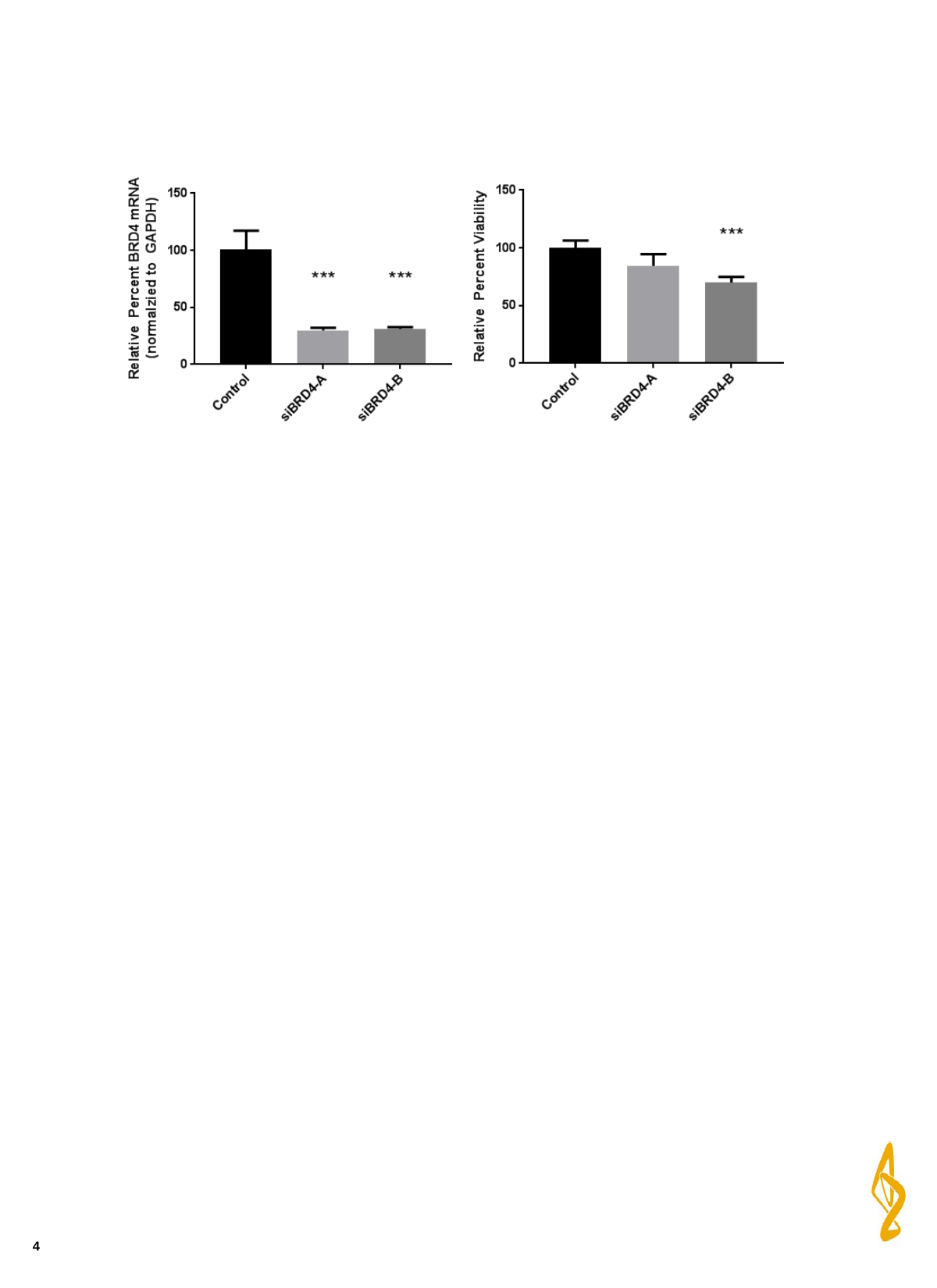

4
